# Mental health and well-being of healthcare workers during the COVID-19 pandemic in the UK: contrasting guidelines with experiences in practice

**DOI:** 10.1192/bjo.2020.148

**Published:** 2020-12-10

**Authors:** Norha Vera San Juan, David Aceituno, Nehla Djellouli, Kirsi Sumray, Nina Regenold, Aron Syversen, Sophie Mulcahy Symmons, Anna Dowrick, Lucy Mitchinson, Georgina Singleton, Cecilia Vindrola-Padros

**Affiliations:** Department of Health Service and Population Research, Institute of Psychiatry, Psychology and Neuroscience, King's College London, UK; and Rapid Research Evaluation and Appraisal Lab, University College London, UK; Department of Health Service and Population Research, Institute of Psychiatry, Psychology and Neuroscience, King's College London, UK; and Department of Psychiatry, School of Medicine, Pontifical Catholic University of Chile, Chile; Institute for Global Health, University College London, UK; and Rapid Research Evaluation and Appraisal Lab, University College London, UK; Institute of Epidemiology and Health Care, University College London, UK; Department of Anthropology, University College London, UK; Institute of Epidemiology and Health Care, University College London, UK; Institute of Epidemiology and Health Care, University College London, UK; Institute of Population Health Science, Queen Mary University of London, UK; Marie Curie Palliative Care Research Department, University College London, UK; Health Services Research Centre, National Institute of Academic Anaesthesia, London, UK; and Rapid Research Evaluation and Appraisal Lab, University College London, UK; Department of Targeted Intervention, University College London, UK; and Rapid Research Evaluation and Appraisal Lab, University College London, UK

**Keywords:** COVID-19, healthcare workers, well-being, qualitative research, rapid review

## Abstract

**Background:**

Substantial evidence has highlighted the importance of considering the mental health of healthcare workers during the COVID-19 pandemic, and several organisations have issued guidelines with recommendations. However, the definition of well-being and the evidence base behind such guidelines remain unclear.

**Aims:**

The aims of the study are to assess the applicability of well-being guidelines in practice, identify unaddressed healthcare workers’ needs and provide recommendations for supporting front-line staff during the current and future pandemics.

**Method:**

This paper discusses the findings of a qualitative study based on interviews with front-line healthcare workers in the UK (*n* = 33), and examines them in relation to a rapid review of well-being guidelines developed in response to the COVID-19 pandemic (*n* = 14).

**Results:**

The guidelines placed greater emphasis on individual mental health and psychological support, whereas healthcare workers placed greater emphasis on structural conditions at work, responsibilities outside the hospital and the invaluable support of the community. The well-being support interventions proposed in the guidelines did not always respond to the lived experiences of staff, as some reported not being able to participate in these interventions because of understaffing, exhaustion or clashing schedules.

**Conclusions:**

Healthcare workers expressed well-being needs that aligned with socio-ecological conceptualisations of well-being related to quality of life. This approach to well-being has been highlighted in literature on support of healthcare workers in previous health emergencies, but it has not been monitored during this pandemic. Well-being guidelines should explore the needs of healthcare workers, and contextual characteristics affecting the implementation of recommendations.

On 11 March 2020, the World Health Organization (WHO) declared COVID-19 a global pandemic.^1^ The rapid spread of this virus, with limited effective treatments currently available, has led to a global crisis with overwhelmed health systems that have had to rapidly adapt to respond to the emergency. This has particularly been the case for the UK, the most affected country in Europe, where, at the time of writing, at least 286 194 people have been infected and 40 542 people have died.^2^

Healthcare systems around the world have been under high demand, with front-line staff exposed to unprecedented strain. Healthcare workers (HCWs) have been facing work overload, fear of infection, frustration, discrimination, isolation and lack of contact with their families.^3^ Substantial evidence from similar extreme situations, including previous epidemics, has highlighted the importance of considering front-line workers’ mental health and well-being.^4^ HCWs work long hours under pressure, often without proper resources, facing inherent dangers and lacking clarity regarding the limits of their duty of care.^5^ Several organisations have issued guidelines with recommendations on how to protect the well-being of HCWs.^6^ However, the definition of well-being and the evidence base behind such guidelines remains unclear. There is a tension in the well-being literature between individual/clinical and ecological approaches, which has implications for the focus of interventions.^7^ The Boorman review, commissioned by the UK Department of Health to address healthcare staff well-being in 2009, and subsequent research derived from it, advocated for a ‘whole-system’ and participatory approach to create well-being programmes.^7,8^ A recent report on well-being of emergency responders in the UK, and a systematic review of the psychological effects of virus outbreaks on HCWs, echoed the importance of assessing organisational factors of well-being, such as shift planning, access to personal protective equipment (PPE) and tensions with colleagues, and external factors affecting mental health, such as stigma and financial or family concerns.^9,10^ These recommendations contrast with a proliferation of surveys assessing only clinical well-being and focusing on reporting the prevalence of mental health problems during the emergence of COVID-19.^11–13^ Critics of the clinical model of well-being have highlighted limitations regarding the lack of sensitivity across contexts and not including outcomes that are meaningful to end-users.^14^ Consequently, proposed guidelines based on this model may fall short when addressing the well-being of HCWs needs in practice, and may not recognise the potential barriers to the implementation of recommendations.

## Aims

This paper discusses the findings of a qualitative study based on interviews with front-line healthcare staff in the UK, and examines them in relation to a rapid review of well-being guidelines developed in response to the COVID-19 pandemic. The aims of the study are to assess the applicability of guidelines in practice, identify unaddressed needs of HCWs and provide recommendations for supporting front-line staff during the current and future pandemics.

## Method

### Rapid synthesis of well-being guidelines

#### Search strategy

We conducted a review of the literature following the Preferred Reporting Items for Systematic Reviews and Meta-Analyses (PRISMA) guidelines.^15^ In view of the quickly evolving situation, we adopted a rapid review methodology, following approaches developed by the WHO and the Cochrane Collaboration for rapid evidence synthesis.^16,17^ A protocol was developed before searching, and is registered in the International Prospective Register of Systematic Reviews (PROSPERO identifier CRD42020183393).

We searched for articles and guidelines providing recommendations on promoting well-being, improving mental health or preventing mental health problems in healthcare staff during the COVID-19 pandemic. The search strategy included terms such as ‘well-being’, ‘mental health’, ‘coping’, ‘healthcare workers’ and ‘COVID’. Searches were conducted in PubMed, EMBASE and PsycInfo, and grey literature searches were conducted through OpenGrey and Tripdatabase. The search was conducted on the 23 April 2020. Additionally, we hand-searched key websites and online databases of government institutions and professional societies in the UK, to identify guidelines that may not have been published elsewhere. Finally, we supplemented our search by cross-referencing the included studies. We included articles written in English, with a focus on the UK setting, although international recommendations were included because of their potential influence on local guidelines and practice. A full description of the search strategy can be found in Supplementary File 1 available at https://doi.org/10.1192/bjo.2020.148.

#### Study selection, data extraction and risk-of-bias assessment

To maintain consistency throughout the study selection, three team members (D.A. and S.M.S.) independently screened titles and abstracts, and then assessed whole texts of eligible articles against the full review inclusion and exclusion criteria. Two researchers (D.A. and S.M.S.) used a data extraction form to extract the data from the guidelines selected for inclusion. The data extraction form was piloted, the required changes were discussed between researchers and the form was revised. We extracted relevant information from the included articles alongside well-being recommendations, such as date, specific healthcare staff groups, definitions of well-being referenced and evidence supporting recommendations. D.A. cross-checked all of the data extraction forms to ensure consistency. We synthesised the extracted data based on recommendations to maintain well-being and prevent mental illness in an aggregative/descriptive manner, summarising information into categories that were common across the included guidelines. Finally, the Reporting Items for Practice Guidelines in Healthcare (RIGHT) tool^18^ was used to appraise the methodological quality of the included guidelines.

### Qualitative study: front-line staff perceptions and experiences of well-being

#### Data collection

This qualitative study is part of a larger ongoing project conducted by the Rapid Research, Evaluation and Appraisal Lab (RREAL), which was designed as a qualitative rapid appraisal with the aim of analysing HCWs’ experiences and perceptions of delivering care during the COVID-19 pandemic.^19^ Rapid appraisals are developed to collect and analyse data in a targeted and iterative way within limited timeframes, and to ‘diagnose’ a situation.^20,21^ The research team combined expertise in mental health and health services research. One of the authors is also an anaesthetist and intensive care doctor, and provided clinical and healthcare organisation and management input. Four of the researchers are senior researchers with years of experience in qualitative research.

A purposive sample of HCWs was selected for interview based on their role in acute care hospitals in the UK. In this paper, we report on the findings from the first 33 interviews carried out with front-line staff between 19 March and 24 April 2020. Potential participants were approached by local investigators with a copy of the participant information sheet to see if a researcher could contact them about taking part in the study. The researcher then contacted via email those who agreed, sending the participant information sheet as well as an interview consent form. Those who agreed to take part in the study were asked to sign the consent form and email it to the researcher.

Semi-structured interviews were carried out by K.S., A.S., L.M., G.S. and C.V.-P. Interviewers received specific training on data security awareness from National Health Service (NHS) Digital and Health Education England. There was no relationship between interviewers and interviewees before the interviews, and correspondence was limited to arranging a time for the interview to take place. Interviews were conducted over the phone, audio-recorded and additional notes on the main topics were documented. The interviews were guided by an interview topic guide. The full interview topic guide can be found in Supplementary File 2. The guide was piloted during the first five interviews, and then revised. It was applied consistently across all interviews, but the order of the questions was guided by the interviewee and adapted to the flow of the conversation.

All personal identifiers from the interview transcripts were removed. Data were kept on a secure server and interviewees were grouped in generic role categories, rather than job title, to avoid individuals being identifiable when quoting from interviews.

The authors assert that all procedures contributing to this work comply with the ethical standards of the relevant national and institutional committees on human experimentation and with the Helsinki Declaration of 1975, as revised in 2008. This study was approved by the Health Research Authority in the UK (Integrated Research Application System: 282069) and the local research and development offices where the study took place. All participants provided consent before taking part.

#### Data extraction and analysis

A group of the authors (N.V.S.J., K.S., N.D., N.R., A.S.) performed selective transcription of extracts from the interviews and interview notes that were related to mental health and well-being. We also included additional information on demographic characteristics of the participants to contextualise the information discussed in the transcripts.

We analysed the data by framework analysis.^22^ We developed an analytical coding framework based on a preliminary scan of the data and inputted it in a Microsoft Excel matrix, with the emerging codes in the columns and cases in rows. The framework was refined during team discussions and all researchers were asked to apply the same framework across their assigned interview transcripts. N.V.S.J. cross-checked the data during the coding process to ensure consistency across researchers. After indexing was completed, we synthesised the key topics emerging within each code, and based on this, developed the final set of themes encompassing the main issues raised by front-line staff. The team also selected quotes from the interview transcripts that could exemplify these themes. N.V.S.J. reviewed the definitions of the themes to ensure consistency in relation to the grouping of codes and the selection of illustrative quotes.

## Results

### Synthesis of well-being guidelines

**Fig. 1:**
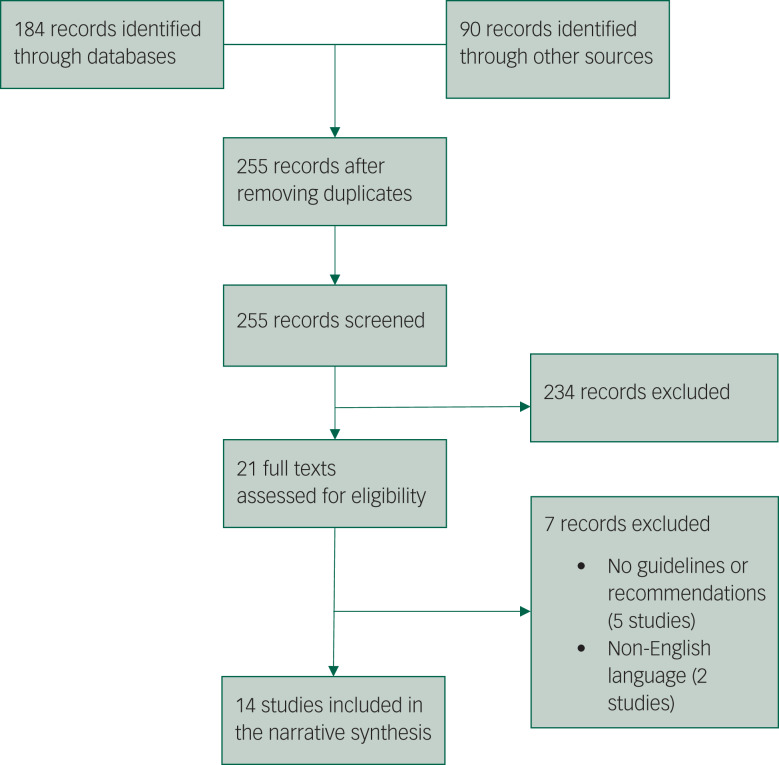
Preferred Reporting Items for Systematic Reviews and Meta-Analyses flowchart.

We identified 255 unduplicated papers/reports from databases and additional sources. After screening titles and abstracts, 21 articles were reviewed in full text. A final 14 articles were included in this review, according to the pre-specified inclusion criteria.^6,23–35^ The PRISMA flowchart showing this process is depicted in Fig. 1.

The main characteristics of the included studies can be found in Supplementary File 3. The studies were published at the peak of the UK pandemic, between March and April 2020. Nine articles consisted of practical recommendations published on institutional webpages and local repositories, and five were journal articles. Guidelines proposed by institutions were developed mostly for the attention of healthcare managers and leaders, whereas the five journal articles were aimed at giving well-being and mental health recommendations directly to HCWs delivering care to patients with COVID-19.

Three reports^28–30^ highlighted that work under pressure and risk of burnout were pre-existing conditions among HCWs (see Table 1). As a result, the guidelines highlighted self-monitoring and help-seeking to prevent more severe mental health problems, and recommended that managers be proactive in detecting mental health concerns among staff and in providing psychological support. We did not find any recommendation to use specific interventions or screening tools to detect mental illness.

**Table 1:** List with main recommendations and their associated guidelines

Recommendations	Guidelines (reference number)
Individual level	
Physical health measures/routine	
Keep your routine	21,22,29
Avoid smoking, drinking and drug use	6,21,25
Get sufficient rest	6,18,21,22
Breaks between shifts	
Sleep at night (routine, screen time, unwind, blackout blinds)	
Eat well	6,21,24
Exercise	6,21
Communication	
Keep in contact with family, friends and colleagues	6,18,22,25,28
Keep informed	16,24,25,28
Raise concerns and ideas for actions	18,24
Peer support	
Be forgiving and patient toward others	18,23,28
Informally support each other emotionally	28,24
Continue training and supervision	24,28
Managing emotions/mindfulness	
Practice mindfulness	22,27
Seek help if you need it	6,22,24,25,28,29
Check in with yourself emotionally and accept this is a difficult time	23
Practice self-care	23,24
Shift to more positive mindset to deal with moral distress	24,25
Managing work burden	
Keep a grounded motivation to work	18
Focus on what you can control, not on what you can't	18
Organisation level	
Covering basic well-being and space needs	
Meals, rest rooms, other location to sleep if need to isolate from family etc.	17,18,26
Communication	
Good-quality communication between colleagues	6,17,20,26,28,29
Facilitate support for staff (follow protocol for assessment and treatment)	24,28,29
Accurate information updates and info on support	6,18
Visible leadership	17,29
Cohesive team: ‘we are in this together’ mentality	19,20,24
Ensure staff raise any concerns they have	19
Encourage self-awareness of staff's emotions	20
Work setting and running services	
More services needed to assist staff when ill	24
Provide training	17,20,26
Rapid ethics committee for end-of-life decisions	24
Flexible working schedules for staff	6,25
Maintain usual activities and services	20
Learn from experiences and do not blame	20,25
Maintain different roles of staff (value staff expertise) and deploy based on staff preferences	17,20
Prepare for recovery phase in advance	20,29
General support	
Ensure peer support	17,18,19,20,24,25,29
Psychological care to patients/their family is good for staff too	17,26
Organise thanks and rewards at recovery phase	17
Debrief and continue to understand support needs	17,19,20,25
Psychological support	
Facilitate access to mental health support	6,17,18,19,24,25,26,27,29
Educate staff on psychological first aid	6,18
Normalise psychological response	17,18,25
Trauma advisors	18
Screen and monitor staff for mental health needs	18,19,29
Ensure psychological interventions are evidence-based	19,29
Recognise that anxiety affects functioning of staff and organisation	20
Act immediately to anxiety	20
Identify staff with high risk for mental problems	20

Guideline recommendations either focused on an individual or institutional/organisational level, with few providing recommendations for both levels. Individual-level recommendations encouraged staff to maintain normal routines, healthy physical and mental health practices, express concerns and keep in contact with family, friends and colleagues. Guidelines highlighted the role of trauma and response to mental health crisis, the importance of allocating time for healthy eating and sleeping habits, attending relevant clinical training to improve self-efficacy and sense of control, and access to psychological therapy and adequate PPE. Guidelines encouraged psychological and peer support; however, some guidelines discouraged single session debriefing because of a possible increased risk of post-traumatic stress disorder (PTSD).^23^ Lastly, practical advice included limiting the consumption of news and social media to avoid feeling overwhelmed by information about the pandemic, and encouraged practicing relaxation and mindfulness techniques.

Organisational-level recommendations focused on necessary institutional arrangements to ensure a positive and supportive environment. Examples of this were recommendations around visible leadership, organising peer support groups, flexibility in working shift patterns, reserving a space for essential well-being practices (eating, sleeping, etc.) and establishing rapid ethics boards to support difficult end-of-life decisions.

The guidelines’ theoretical assumptions around the concept of well-being and how this is affected by the pandemic were stated in ten articles. One assumption regarding well-being was based on a psychosocial resilience approach,^28^ referencing a framework that focused on individuals maintaining social ties through online platforms and managers reinforcing team-working.^36^ Nine guidelines based their recommendations on the individual's potential to develop mental illness, in particular PTSD, and individual needs emerging from exposure to the virus or difficult life and death decisions. Four guidelines did not specify any conceptual framework or definition of well-being to inform their recommendations.

The quality appraisal based on the RIGHT tool yielded heterogeneous results. Most guidelines were clear about the aims, the target population and the context where the recommendations ought to be applied. Well-being recommendations were generally precise, and some were formulated in a manner that allowed the development of actionable measures. The guidelines provided little detail of the evidence behind their suggestions and none specified the certainty or strength of their recommendations. They also failed to provide explicit practical recommendations for implementation. A detailed analysis with the RIGHT tool can be found in Supplementary File 4.

### Qualitative study: front-line staff perceptions and experiences

The main characteristics of the sample have been included in Table 2.

**Table 2:** Characteristics of the study sample

Variables		Number of participants
Gender	Male	13
	Female	20
Professional groups	Nurses	3
	Anaesthetists	19
	Other doctors	9
	Allied health professionals	2
Level of seniority	Senior	21
	Junior	12

Five themes emerged from the qualitative interviews: well-being and ‘pulling together’; concerns, unsettling experiences and difficult moments; experiences around PPE; morale and barriers to performing confidently; and life outside the clinical role. The text below defines each theme and includes illustrative quotes. Supplementary File 5 provides an overview of key characteristics and additional illustrative quotes for each theme.

#### Well-being support and ‘pulling together’

There was a significant focus on HCWs’ mental health and psychological support as part of service adaptations during the pandemic. Examples of this were increasing the availability of clinical psychologists to provide therapy, online counselling or group support sessions. However, staff reported that additional workload or clashing schedules prevented them from participating in these formal well-being activities:

‘Part of the problem for the official support, there is a psychologist who's offering sessions, but they are in the middle of the day. So, you wouldn't be able to go if you were on nights, or if you are clinically busy you can't really attend that in the middle of the shift […], but informal peer-support groups are starting which has been quite good.’ (Anaesthetist 07).

Some staff mentioned there was ‘no room’ for well-being sessions during the peak of the pandemic, and they prioritised resting during breaks.

The most salient form of well-being support mentioned by staff was mutual moral and clinical support between HCWs. This support happened through buddy systems and spontaneously, as a general feeling of motivation, comradery and empowerment among teams. Factors that were said to contribute to positive team dynamics were a less hierarchical structure and maintaining consistency in the composition of working teams. However, positive group dynamics between colleagues could be negatively affected by the lack of clarity around duty of care between different medical specialties and the lack of testing for HCWs. This last point was considered woefully inadequate and distressing for staff and their families, as well as exacerbating guilt from being off work without a ‘justified’ cause.

HCWs felt appreciated and valued as a result of community support, citing examples such as rainbow pictures and clapping. Food donated by local restaurants and neighbours was reported to have a significant impact on keeping morale high and getting staff through long shifts.

#### Concerns, unsettling experiences and key difficult moments

Participants described a range of struggles at different stages of the pandemic. In the pre-peak and preparation stages, HCWs experienced anxiety and anticipation mainly in relation to news reports of international experiences in intensive care units (ICU); the possibility of bringing the virus home to families; and moving into a ‘greatest good for the greatest number’ model of care (where decisions need to be made about the rationing of scarce services between patients), which could have moral and legal implications for clinicians. Worries were most alleviated through training, gaining experience in new roles and transparent and consistent communications from managers. In general, staff reported that anxiety diminished as they became more immersed in their work and, in some cases, once they had contracted and recovered from the virus. Those who worked in particularly busy departments expressed anxiety about understaffing owing to sickness when more patients with COVID-19 started presenting.

During the height of the pandemic, staff mentioned experiencing additional cognitive burdens to their usual work, such as uncertainties around diagnosis without the usual diagnostic techniques available and feeling overwhelmed by frequent changes in PPE and clinical guidelines. At the same time, staff spoke about additional moral labour to their usual ICU work because patients’ families were not allowed to visit. Two moments identified as particularly difficult in this respect: when patients were initially taken into the ICU and when patients were dying. HCWs spoke of difficulties interacting with the families of dying patients remotely:

‘When they are getting critically ill and we're communicating with the family on the phone rather than seeing the family face-to-face that's just the other aspect of managing patients with COVID which I think all the doctors and nurses have found really difficult and really challenging.’ (Doctor 33).

As the peak passed, concerns shifted toward the backlog of patients without COVID-19 who had not received care during this period, and fears regarding how staff and patients would cope with a second peak.

#### Experiences around PPE

PPE was a consistent challenge for all HCWs. During the early stages of the pandemic, HCWs experienced anxiety in relation to the correct donning and doffing procedures for PPE. Later in the pandemic, working in full PPE and not having proper breaks was mentioned as a factor generating great distress. Staff reported overheating, dehydration and exhaustion, and expressed discomfort owing to PPE being ‘one size fits all’ and thus causing pain or feeling claustrophobic. Rapidly changing and conflicting guidelines on PPE use caused anxiety. Many HCWs worried guidelines were changing because of lack of stock rather than safety standards.

Staff emphasised working in full PPE as a factor that hindered communication with colleagues and the generation of a close relationship with patients and their families. In particular, staff mentioned that ‘everything came out as a shout’, and it was not possible to identify colleagues behind masks:

‘The other anxiety is, I am managing patients in an intensive care environment which is not an environment that I'm used to working with, I don't know any of the people working on ICU. I don't know the nurses, the junior doctors, I don't know the intensivist, and none of them can communicate with me properly because I'm wearing a mask’ (Anaesthetist 25).

Staff expressed ambivalence about how families should have been involved in patient care, questioning whether it was appropriate for them to visit patients and wear PPE. The possibility of patients dying alone generated great distress for HCWs, patients and families. Some HCWs felt that permitting family to accompany dying patients should be a priority. Others commented on feeling nervous about large families making use of limited stocks of PPE before regulations began.

#### Morale and barriers to performing confidently

Staff reported morale was generally high; they felt positive changes to services had happened efficiently, senior management were responsive and junior doctors and nurses felt empowered as their suggestions were being heard:

‘I think most trainees feel that it is very easy to make suggestions on how things can be improved, and I think the more senior clinicians and management team have been quite receptive, have taken many of these on board’ (Anaesthetist 05).

However, participants expressed concerns that morale may deteriorate as weeks went by working under strenuous conditions. An important barrier to performing confidently was lack of sleep owing to increased workload to cover staff sickness and an increment in the number of night shifts that staff were required to work.

Challenges varied between professional groups; junior doctors faced uncertainty regarding examinations and courses that had been paused, and consultants were required to manage much larger teams and keep up to date with frequently changing protocols. Some doctors working with patients with COVID-19 found their self-efficacy was weakened by lack of clarity regarding clinical protocols.

ICU nurses were thought to have had some of the largest increases in workload and to be emotionally affected by not being able to provide the quality of work they were used to. Another significantly affected group were anaesthetists, who had to rapidly take on new roles as intensivists, working alongside new colleagues and under the constraints of PPE.

#### Life outside the clinical role

The professional experiences of HCWs during the pandemic were greatly influenced by their lives outside of work. Some staff reported initially experiencing negative interactions with neighbours or childminders who perceived them to be a greater risk of infection. However, for the most part, participants expressed feeling incredibly fortunate and grateful for the community support.

The main concerns outside the hospital were caring for children and elderly relatives, completing shopping and housework, and arranging their travel to and from the hospital:

‘I think people who live by themselves are finding it very difficult, because they are having to think ahead and make sure to prepare food on their days off so that, on their days on, they have something prepared in the fridge or the freezer.’ (Anaesthetist 14).

Navigating caring duties proved complicated; supportive families and sustained schooling provided key relief:

‘Our childminder […] decided 2 weeks ago that because we are healthcare workers they didn't want to look after my kids anymore. The school then shut […]. I had a text from one of the other mums at the nursery, […] she wasn't sure she would want her child to go in view of the fact my other half and I both work at the hospital. Both of these events really affected me and my ability to focus at work.’ (Anaesthetist 06).

Staff appreciated the senior management team planning to cover needs beyond HCW clinical work, such as providing free parking to facilitate travel to and from work, optional accommodation and hospital child care remaining available. Also helpful were bicycle and transport providers offering free rides for NHS workers.

Physical spaces, such as ‘wobble’ rooms and health hubs, were reported to provide much-needed areas to rest and to recharge. However, working facilities varied across sites. Some staff described few spaces for resting and a lack of cleaning facilities and showers to prevent contamination.

HCWs mentioned the increased difficulty of maintaining a life–work balance because of the combination of increased workload and the limited number of leisure options available. People who lived alone found isolation particularly difficult and some reported being expected to do significantly more clinical work to cover for colleagues who had family responsibilities. Isolation was also particularly difficult for people with pre-existing mental health conditions or difficult situations at home.

## Discussion

### Mapping guideline themes and staff experiences

Findings in this study make apparent the significant concern and effort made by mental health professionals to respond rapidly to the mental health needs of HCWs at very early stages of the pandemic. A high awareness of mental health and well-being needs was demonstrated by the number of guidelines available to safeguard and support HCWs as the pandemic reached its peak. Guidelines as a whole tended to concur with staff's concerns around individual and organisational factors affecting well-being. However, there was discordance in terms of where emphasis was placed, and important gaps in guidelines relating to external factors such as the role of community support, and barriers to materialise the recommendations in practice. Key recommendations for guideline development are outlined in Table 3.

**Table 3:** Recommendations for guideline development

• Base recommendations on holistic understandings of well-being or clarify which aspects of well-being are being addressed and possible limitations of this. • Consider basic well-being priorities such as sleeping, eating and access to protective equipment. • Consider the role of external factors, such as community support or stigma toward healthcare workers, caring responsibilities and effective policy responses. • Only provide evidence-based recommendations, making use of the significant number of official reports, systematic literature reviews and longitudinal studies available. • Consider contextual barriers that may hinder the implementation of recommendations and propose alternatives. • Involve staff at all stages of guideline development.

As a whole, the guidelines placed greater emphasis on well-being at an individual level. This was under the premise that staff were likely to develop mental disorders as a result of having to make difficult life or death decisions or being exposed to the virus without appropriate PPE. This clinical approach to well-being speaks of mental distress in terms of diagnosis, and frames well-being in terms of outcome measures of symptoms and psychosocial functioning designed by mental health professionals.^37,38^ To address the mental health of HCWs during emergencies, research suggests that a more holistic approach, aligned with socio-ecological conceptualisations of well-being, is required.^7,11,39,40^

Responses from staff in this study echoed those of HCWs in China during COVID-19 and the results from the systematic review regarding the psychological effects of virus outbreaks on HCWs.^3,10^ Staff placed greater emphasis on structural conditions at work, such as understaffing, adequate rest, meals and access to PPE, and other broader factors influencing their well-being, such as the invaluable support of the community and the legal implications of their work. Research from previous virus outbreaks pointed to the role of community support as being a key contextual facilitator of well-being.^41^ In this study, staff described an important shift in community attitudes toward HCWs; staff and their families were initially stigmatised, but later admired and rewarded by the community. These findings suggest that implementing ‘appreciation’ and anti-stigma campaigns to promote support to HCWs should be included as a key preparation to support staff's well-being.^42^

At the same time, the need to take a holistic approach to planning well-being aid in emergencies has been highlighted in emergency responses to other epidemics, such as modern approaches to HIV prevention, which are directed toward addressing structures that constrain or enable people's choices.^39^ A focus on mental illness, and PTSD in particular, has been criticised in past mental health emergency interventions, citing its lack of attention to practical well-being needs and potential misconceptions of healthy individual responses.^43–45^ An example of this in our study was a guideline suggesting that staff who repeatedly expressed not being available to attend peer support groups should be interpreted as using ‘avoidance’, and it being a potential symptom of trauma; staff, on the other hand, reported having very few breaks because of understaffing and wishing to dedicate these breaks to resting rather than engaging in support groups. Additional ambiguity in interpretations can be expected to arise because of a lack of recommendations in guidelines around specific interventions or screening tools to detect mental illness.

Discrepancies between what staff perceived as important and what was prioritised in well-being guidelines highlight that it is imperative to base guidelines on available high-level evidence, such as the systematic reviews and longitudinal research designs referenced in this text. Alongside this, involving staff at all stages of developing well-being support strategies to respond to local needs and contexts is paramount. The latter was one of the five key recommendations of the Boorman review.^8^ Situating practice in its context and combining organisational, community and personal strategies for well-being has shown to increase the likelihood of success in well-being promotion interventions.^46,47^

An organisational factor that stood out in guidelines and the qualitative findings was HCWs’ feelings around being stronger working as a collective and ‘pulling together’ in less hierarchical working conditions, while at the same time maintaining strong leadership, guidance and sustained open communication from managers. This is recognised in literature about healthcare staff well-being, which states that leadership styles focused on transparency, consistency and empowerment of staff lead to higher employee engagement and work satisfaction.^47^ However, results from the qualitative study showed senior staff faced great difficulty in keeping up their supporting roles because of having to manage larger teams composed of people they may not have worked with previously; no guidelines dealt with this matter. Additional research is needed to ascertain the toll of additional responsibilities on staff performing leadership roles and potential strategies to address this. Gaggioli and Riva^48^ propose that using apps to monitor individual well-being is a strategy that is more flexible and can accommodate staff needs, while at the same time reducing the time required for managers and staff to communicate about this. The use of apps could be a potential strategy to help managers deal with the increase in responsibility and to give staff more opportunities to engage in well-being activities at a time suitable for them; it may also help to integrate and implement individual- and organisational-level mental health guidelines.

### Study strengths and limitations

The findings of this study should be considered in light of its strengths and limitations. The rapid review and qualitative study were conducted by a multidisciplinary team, following strong systematic research methods and guidelines, and allowed for a rich discussion contrasting both sources of data to reveal gaps between guidelines and practice. The rapid design allowed us to share findings in a timely way. Applicability of the findings outside the context of this study should be evaluated, taking into consideration the rapidly changing circumstances of the current health emergency. New well-being guidelines may emerge later in the pandemic, or changes affecting HCWs experiences may occur, which we have not captured. Sensitive topics relevant to well-being, such as alcohol or drug consumption, were not present in participants’ answers. This paper focuses on the first 33 interviews of our study, a sample that includes a higher proportion of women, doctors, senior staff and White ethnicity, leaving perspectives from other groups unexplored. Furthermore, the study did not map the local offer of well-being support resources, and was only based on participants’ perceptions of well-being support. Because of the rapid analysis process used in this study, we were not able to integrate a member checking phase and relied on internal cross-checking strategies. Member checking is being used in the wider study.

To the authors’ knowledge, this is the first study contrasting well-being guidelines and staff experiences in practice during the COVID-19 emergency. The findings in this study extend the understanding of well-being guidelines in the light of HCWs’ experiences in practice. Well-being guidelines and further research on this topic should go beyond focusing on top-down clinical understandings of well-being, to also explore staff's needs and the contextual characteristics affecting the implementation of recommendations. Guidelines need to provide practical methods for implementation and propose flexible psychological support, to avoid ambiguity at an organisational level. Future HCW mental health emergency responses should incorporate participatory approaches that allow for the development of responsive and adaptative well-being programmes.

## Data Availability

All data relevant to the study are included in the article or uploaded as Supplementary material. The lead author affirms that this manuscript is an honest, accurate and transparent account of the study being reported; that no important aspects of the study have been omitted; and that any discrepancies from the study as planned (and, if relevant, registered) have been explained.

## Supplementary material

For supplementary material accompanying this paper visit https://doi.org/10.1192/bjo.2020.148. click here to view supplementary material
