# Supplementary material for: Mental health and well-being of healthcare workers during the COVID-19 pandemic in the UK: contrasting guidelines with experiences in practice
Source: BJPsych Open. 2020 Dec 10;7(1):e15. doi: 10.1192/bjo.2020.148 (PMC7844154; doi:10.1192/bjo.2020.148)
Supplement: Supplementary file 1 [file S2056472420001489sup.zip › S2056472420001489sup001.zip!/Supplementary_material_1_PROSPERO_protocol_(1).docx]

Supplementary material 1

## Guidelines and workplace interventions to improve wellbeing and reduce mental health problems of healthcare workers during the COVID-19 pandemics – Search strategy

## Criteria for considering studies for this review

We will search published and unpublished studies and guidelines specifically designed to promote healthcare wellbeing and prevent mental health problems. We will use the following criteria to select the studies:

|  | Inclusion | Exclusion |
| --- | --- | --- |
| Participants | Healthcare workers involved in the care of patients affected by COVID-19. | Any other professional outside the healthcare sector. |
| Intervention | Guidelines and recommendations specifically designed to promote wellbeing or protect staffs’ mental health (delivered in any format). | Guidelines specifically designed to embrace COVID-19 pandemics with no wellbeing or mental health components (e.g.: ventilation recommendations).  Generic guidelines to promote wellbeing outside the epidemiological emergency. |
| Comparator | N/A |  |
| Outcomes | Wellbeing promotion, mental health promotion, mental illness prevention. | Behavioural change aimed to manage the pandemic or other health outcome. |
| Study design | Randomised-controlled trials, non-randomised trials, observational studies, clinical guidelines | Modelling studies |
| Setting | United Kingdom |  |

### Search methods for identification of studies

We will search for articles published in academic peer reviewed journals using the following databases: PubMed, EMBASE and PsycInfo. We will identify additional articles by cross-referencing the included articles.

In addition, we will carry out a grey literature search by using the following sources:

**Source**

WHO webpage <https://search.bvsalud.org/global-literature-on-novel-coronavirus-2019-ncov/>

British Psychological Society <https://www.bps.org.uk/>

Royal College of Psychiatrists <https://www.rcpsych.ac.uk/>

National Institute for Health and Care Excellence (NICE) <https://www.nice.org.uk/guidance/published?type=cov,coa>

Pre-prints:

- MedRxiv: <https://www.medrxiv.org/>
- PsyArxiv: <https://psyarxiv.com/>

Grey literature

- Trip database: <https://www.tripdatabase.com/>

- <http://www.opengrey.eu/>

Cross-referencing

Finally, considering the ongoing nature of the situation, we assume many interventions taking place are not necessarily available on official sources. Therefore, we will contact experts in the field and local health authorities.

Search strategies:

**Medline (Ovid ®)**

Search terms:

1. wellbeing.mp.
2. exp Mental Health/
3. exp Mental Disorders/
4. mental illness.mp.
5. coping
6. exp Adaptation, Psychological/
7. exp Self Care/
8. self-care.mp.
9. covid.mp.
10. exp Coronavirus Infections/
11. sars-cov-2.mp.
12. exp Health Personnel/
13. healthcare workers.mp.
14. or/1-8
15. or/9-11
16. or/12-13
17. 14 and 15 and 16

**Embase (Ovid ®)**

Search terms:

1. exp wellbeing/
2. exp mental health/
3. mental disorders.mp.
4. exp mental disease/
5. coping.mp
6. self-care.mp.
7. exp self care/
8. covid.mp.
9. 2019 nCoV.mp.
10. novel coronavirus.mp.
11. exp Coronavirinae/
12. exp SARS coronavirus/
13. exp health care personnel/
14. exp health practitioner/
15. healthcare workers.mp.
16. or/1-7
17. or/8-12
18. or/13-15
19. 16 and 17 and 18

**PsycInfo (Ovid ®)**

Search terms:

1. exp Well being/
2. exp Mental Health/
3. exp Mental Disorders.mp.
4. exp Coping Behavior/
5. coping.mp.
6. exp Self-Care Skills/
7. selfcare.mp.
8. covid.mp.
9. 2019 nCoV.mp.
10. novel coronavirus.mp.
11. exp Pandemics/
12. exp health personnel/
13. healthcare workers.mp.
14. or/1-7
15. or/8-11
16. or/12-13
17. 14 and 15 and 16

After retrieving results, these will be imported into Rayyan®, a web-based collaborative screening tool [1]. After deduplication, two authors will screen titles and abstracts independently. Any disagreement will be resolved by discussion with a third team member. Full-texts considered after titles and abstracts screening will be reviewed independently by the same reviewers and contrasted against inclusion and exclusion criteria.

### Data collection and analysis

Data from the included studies will be extracted by two members of the team independently. Data will be extracted using pilot-tested data extraction forms in Microsoft Excel® and it will include the following items:

**Authors**

**Title**

**Date**

**Institution**

**Setting**

**Target population(s)**

**Wellbeing definition**

**Mental Health definition**

**Mental illness definition**

**Conceptual framework**

**Evidence behind**

**Organization-level recommendations**

**Individual-level recommendations**

**Further help resources**

Furthermore, quality assessment of the included studies will be applied using different tools depending on the study design. For instance, in the case of randomised-controlled trials (RCT), we will use the Cochrane Risk of bias tool, meanwhile for those non-randomised trials we will use the

Risk Of Bias In Non-Randomized Studies - of Interventions (ROBINS-I) tool. Clinical guidelines and recommendations will be assessed using the Reporting Items for Practice Guidelines in Healthcare (RIGHT) instrument. Risk of bias will be assessed independently by two authors and differences will be set by consensus or by calling a third reviewer if this is not achieved.

The data extracted will be stored and managed in University cloud services. Because of the data included in this review will be publicly available, we do not anticipate confidentiality concerns to be addressed in the data management.

We will provide a narrative synthesis of the findings from the included studies. Given that we expect a heterogeneous group of studies using different methodologies and outcomes we do not anticipate it will be possible to collate data across studies. However, if more than 4 studies per outcomes is obtained, we will pool effect estimates employing a random-effects meta-analysis, applying the DerSimonian and Laird method [2], anticipating considerable heterogeneity, as previously mentioned. Data assumptions will be checked before conducting such analyses.

### Declarations of Interest

The authors declare no competing interest.

### References

1	Ouzzani M, Hammady H, Fedorowicz Z, *et al.* Rayyan-a web and mobile app for systematic reviews. *Syst Rev* 2016;**5**:210.

2	DerSimonian R, Laird N. Meta-analysis in clinical trials. *Control Clin Trials* 1986;**7**:177–88.
