# Supplementary material for: Mental health and well-being of healthcare workers during the COVID-19 pandemic in the UK: contrasting guidelines with experiences in practice
Source: BJPsych Open. 2020 Dec 10;7(1):e15. doi: 10.1192/bjo.2020.148 (PMC7844154; doi:10.1192/bjo.2020.148)
Supplement: Supplementary file 1 [file S2056472420001489sup.zip › S2056472420001489sup001.zip!/Supplementary_material_3_Included_guidelines_characteristics.docx]

**Supplementary material 3**

**Characteristics of the included studies.**

| **Guideline** | **Authors/Institution** | **Setting** | **Target Population** | **Conceptual framework** | **Evidence-based** |
| --- | --- | --- | --- | --- | --- |
| Mental health and psychosocial considerations during the COVID-19 outbreak | World Health Organization | Global | General population, healthcare workers, managers in health facilities, carers of children, older adults and people with underlying conditions, people in isolation | Psychological response to the crisis/stress | Not stated |
| The psychological needs of healthcare staff as a result of the Coronavirus pandemic | British Psychological Society Covid19 Staff Wellbeing Group | UK | Leaders and managers of healthcare services | Not clearly defined | Not stated |
| Staff wellbeing during the COVID-19 pandemic Guidance for organisations | Tees, Esk and Wear Valleys NHS Foundation Trust | UK | Healthcare organisations leaders and management | Trauma informed approach | Emerging lessons from China’s covid-19 management and previous literature |
| Guidance for planners of the psychosocial response to stress experienced by hospital staff associated with COVID: Early Interventions | COVID Trauma response working group rapid guidance | UK | Planners, managers and team leaders | Trauma informed approach | Consensus of trauma clinicians and researchers |
| Organisational Wellbeing During the Covid-19 Pandemic: A Guidance Document | Medical Psychotherapy Faculty Royal College of Psychiatrists | UK | All staff within a Mental Health Trust | Psychological response to the crisis/stress | Principles drawn from organisational consultancy models can support Mental Health Trusts during times of crisis. |
| Sleep in the time of COVID-19: Advice for NHS Staff | Dr Mike Farquhar  Consultant in sleep medicine at Evelina London Children’s Hospital | UK | NHS staff | Not clearly defined | Not stated |
| Staff Health and Wellbeing Guide - COVID-19 | Mid and South Essex University Hospitals Group | UK | NHS hospital staff | Not clearly defined | Previous literature on mindfulness. |
| Staying Safe & Well: A Self Care Guide for Staff looking after patients with Coronavirus | North of Scotland Trauma Network | UK | NHS staff | Theories of personal and collective psychosocial resilience | Not stated |
| The top ten messages for supporting healthcare staff during the COVID-19 pandemic | Multidisciplinary team from academia, consultancy and the Royal College of Psychiatrists | UK | NHS leaders and clinical and general managers | Theories of personal and collective psychosocial resilience | Previous literature of health, clinical and social psychology and psychiatry. |
| Caring for the carers: Ensuring the provision of quality maternity care during a global pandemic | Wilson AN, Ravaldi C, Scoullar MJL, Vogel JP, Szabo RA, Fisher JRW, Homer CSE. | Australia | Maternity providers and new-born care units' staff, including doctors, midwives and nurses. | Trauma informed approach | National Centre for PTSD (US Department of Veterans Affairs).  CDC and WHO guidelines |
| Preserving mental health and resilience in frontline healthcare workers during COVID-19 | Santarone K, McKenney M, Elkbuli A | USA | Frontline healthcare workers, in general. | Trauma informed approach | Previous literature and ongoing reports from China and Italy |
| Sustainable practice of ophthalmology during COVID-19: challenges and solutions | Lim LW, Yip LW, Tay HW, Ang XL, Lee LK, Chin CF, Yong V | Singapore | Ophthalmology practitioners attending COVID-19 patients | Psychological response to the crisis/stress | 2003 SARS outbreak experience and previous literature |
| Clinician Wellness During the COVID-19 Pandemic: Extraordinary Times and Unusual Challenges for the Allergist/Immunologist | Bansal P, Bingemann TA, Greenhawt M, Mosnaim G, Nanda A, Oppenheimer J, Sharma H, Stukus D, Shaker M | USA | Allergy and Immunology physicians | Psychological response to the crisis/stress | Previous infectious outbreaks, evidence from burnout and grief literature. |
| Managing mental health challenges faced by healthcare workers during covid-19 pandemic | Greenberg N, Docherty M, Gnanapragasam S, Wessely S | UK | Healthcare workers in general | Trauma informed approach | Previous evidence, fundamentally from PTSD literature. |
