# Supplementary material for: Mental health and well-being of healthcare workers during the COVID-19 pandemic in the UK: contrasting guidelines with experiences in practice
Source: BJPsych Open. 2020 Dec 10;7(1):e15. doi: 10.1192/bjo.2020.148 (PMC7844154; doi:10.1192/bjo.2020.148)
Supplement: Supplementary file 1 [file S2056472420001489sup.zip › S2056472420001489sup001.zip!/Supplementary_material_4_RIGHT_quality-appraisal.docx]

**Supplementary material 4. Quality appraisal based on the RIGHT tool**

| **Item** | **1** | **2** | **3** | **4** | **5** | **6** | **7** | **8** | **9** | **10** | **11** | **12** | **13** | **14** |
| --- | --- | --- | --- | --- | --- | --- | --- | --- | --- | --- | --- | --- | --- | --- |
| **Basic information** | | | | | | | | | | | | | | |
| Identification | yes | yes | yes | yes | yes | yes | no | yes | yes | no | no | no | no | no |
| Date | yes | yes | no | yes | no | no | no | yes | yes | yes | yes | yes | yes | yes |
| Focus | yes | yes | yes | yes | yes | no | no | yes | yes | yes | yes | yes | yes | yes |
| **Background** | | | | | | | | | | | | | | |
| Brief description of the problem(s) | yes | no | yes | yes | yes | no | no | no | no | yes | yes | yes | yes | yes |
| Aim(s) of the guideline | yes | yes | yes | yes | yes | no | no | no | yes | yes | yes | yes | yes | yes |
| Target population(s) | yes | yes | yes | yes | yes | yes | yes | yes | yes | yes | yes | yes | yes | yes |
| End users | yes | yes | yes | yes | yes | yes | yes | yes | yes | yes | yes | yes | yes | yes |
| Settings | yes | yes | yes | yes | yes | no | no | yes | yes | yes | yes | yes | yes | yes |
| Guideline development group | no | no | no | no | no | no | no | no | no | no | no | no | no | yes |
| Authors | no | yes | yes | yes | yes | no | no | no | yes | yes | yes | yes | yes | yes |
| **Evidence** | | | | | | | | | | | | | | |
| Health care questions | no | no | yes | no | yes | no | no | no | no | no | no | no | no | no |
| Systematic reviews | no | no | no | yes | no | no | no | no | yes | no | no | no | no | no |
| Certainty of the body of evidence | no | no | no | no | no | no | no | no | no | no | no | no | no | no |
| **Recommendations** | | | | | | | | | | | | | | |
| Recommendations | yes | yes | yes | yes | yes | yes | yes | yes | yes | yes | yes | no | yes | yes |
| Strength of recommendations | no | no | no | yes | no | no | no | no | no | no | no | no | no | no |
| Rationale/explanation for recommendations | no | no | yes | no | no | no | no | no | no | yes | yes | yes | yes | no |
| Evidence to decision processes | no | no | yes | no | no | no | no | no | no | no | no | no | no | no |
| **Review and quality assurance** | | | | | | | | | | | | | | |
| External review | no | no | no | no | no | no | no | no | no | no | no | no | no | yes |
| Quality assurance | no | no | no | no | no | no | no | no | no | no | no | no | no | no |
| **Funding, declaration and management of interests** | | | | | | | | | | | | | | |
| Funding source(s) and role(s) of the funder | no | no | no | no | no | no | no | no | no | no | no | no | no | yes |
| Role of funders | no | no | no | no | no | no | no | no | no | no | no | no | no | yes |
| Declaration and management of interest | no | no | no | no | no | no | no | no | no | yes | no | yes | yes | yes |
| **Other information** | | | | | | | | | | | | | | |
| Suggestions for further research | no | no | no | yes | no | no | no | no | no | yes | no | no | no | yes |
| Limitations of the guideline | no | no | no | yes | no | no | no | no | no | no | no | no | no | no |

1. World Health Organization. Mental health and psychosocial considerations during the COVID-19 outbreak. World Health Organization; 2020 Mar.
2. British Psychological Society Covid19 Staff Wellbeing Group. The psychological needs of healthcare staff as a result of the Coronavirus pandemic. British Psychological Society; 2020.
3. Kennedy A. Staff wellbeing during the COVID-19 pandemic: Guidance for organisations. Tees, Esk and Wear Valleys NHS Foundation Trust; 2020.
4. COVID Trauma response working group rapid guidance. Guidance for planners of the psychosocial response to stress experienced by hospital staff associated with COVID: Early Interventions. University College London & Traumatic Stress Clinic; 2020 Apr.
5. O’Reilly J, Eyres M, Vrabtchev S, Heyland S. Organisational Wellbeing During the Covid-19 Pandemic:A Guidance Document. Royal College of Psychiatrists; 2020.
6. Farquhar M. Sleep in the time of COVID-19: Advice for NHS Staff. 2020. Available: <https://www.aomrc.org.uk/wp-content/uploads/2020/04/Sleep-in-the-Time-of-COVID19-by-Dr-Mike-Farquhar-Consultant-Children-Sleep-Medicine-.pdf>
7. Mid and South Essex University Hospitals Group. Staff Health and Wellbeing Guide - COVID-19. NHS Mid and South Essex University Hospitals Group; 2020.
8. Taylor L, Anderson J. Staying Safe & Well: A Self Care Guide for Staff looking after patients with Coronavirus. NHS Scotland; 2020 Mar.
9. Williams R, Murray E, Neal A, Kemp V. Top ten messages for supporting healthcare staff during the COVID-19 pandemic. Royal College of Psychiatrists; 2020. Available: <https://www.rcpsych.ac.uk/docs/default-source/about-us/covid-19/top-ten-messages-williams-et-al.pdf?sfvrsn=990e3861_0>
10. Wilson AN, Ravaldi C, Scoullar MJL, Vogel JP, Szabo RA, Fisher JRW, et al. Caring for the carers: Ensuring the provision of quality maternity care during a global pandemic. Women Birth. 2020. doi:10.1016/j.wombi.2020.03.011
11. Santarone K, McKenney M, Elkbuli A. Preserving mental health and resilience in frontline healthcare workers during COVID-19. Am J Emerg Med. 2020. doi:10.1016/j.ajem.2020.04.030
12. Lim LW, Yip LW, Tay HW, Ang XL, Lee LK, Chin CF, et al. Sustainable practice of ophthalmology during COVID-19: challenges and solutions. Graefes Arch Clin Exp Ophthalmol. 2020. doi:10.1007/s00417-020-04682-z
13. Bansal P, Bingemann TA, Greenhawt M, Mosnaim G, Nanda A, Oppenheimer J, et al. Clinician Wellness During the COVID-19 Pandemic: Extraordinary Times and Unusual Challenges for the Allergist/Immunologist. J Allergy Clin Immunol Pract. 2020. doi:10.1016/j.jaip.2020.04.001
14. Greenberg N, Docherty M, Gnanapragasam S, Wessely S. Managing mental health challenges faced by healthcare workers during covid-19 pandemic. BMJ. 2020;368: m1211.
