# Supplementary material for: Mental health and well-being of healthcare workers during the COVID-19 pandemic in the UK: contrasting guidelines with experiences in practice
Source: BJPsych Open. 2020 Dec 10;7(1):e15. doi: 10.1192/bjo.2020.148 (PMC7844154; doi:10.1192/bjo.2020.148)
Supplement: Supplementary file 1 [file S2056472420001489sup.zip › S2056472420001489sup001.zip!/Supplementary_material_5_-_Theme_description_and_quotes_(1).docx]

**Supplementary material 5. Findings of the qualitative study and illustrative quotes.**

| **Theme summary** | **Illustrative quotes** |
| --- | --- |
| **Theme 1: Wellbeing and “pulling together”** | |
| - Increase in mental health and psychological support for HCWs - Support services difficult to access for HCWs due to additional workload, clashing schedules, reserving breaks for rest - Wellbeing support found among colleagues, with less hierarchical structure contributing to feelings of motivation, comradery and empowerment - Positive group dynamics negatively affected by the lack of clarity around duty of care between different medical specialties and the lack of testing for HCWs - HCWs felt appreciated and valued owing to community support - HCWs valued physical spaces provided for rest | *“Part of the problem for the official support, there is a psychologist who’s offering sessions, but they are in the middle of the day. So, you wouldn’t be able to go if you were on nights, or if you are clinically busy you can’t really attend that in the middle of the shift […], but informal peer-support groups are starting which has been quite good.”* (Anaesthetist 07)  *“I am worried there is not enough provision on wards for end of life care […]. This is going to have an enormous emotional effect on those working there, especially young newer colleagues. The health hub is there, but how confident are people in accessing it?”* (Nurse 17)  *“Things would be much easier if we could get tested right away because we could know if you have to come into work or stay at home. […] it was very anxiety provoking not knowing. […] When you are sick it’s awful because […] you’re talking about 8 of your friends who you know are suffering as well having to cover you and you know who and you see the WhatsApp messages of them negotiating who is gonna cover your shift, it’s really heart breaking. I think if you could say ‘I have a diagnosis of coronavirus, it’s gonna be 7 days’ […] I just think you wouldn’t feel so desperately guilty about putting your colleagues out.”* (Anaesthetist 25)  *“The biggest failing in the UK is not testing workers. My son has a fever and we are all stuck at home for two weeks without knowing if we have the virus or not. […] I want to follow guidance, but I know I am also needed on the ward."* (GP 02)  *“I think that’s been a really big problem. I almost certainly had COVID myself, I became unwell on a Friday afternoon and went home. I couldn’t be tested before I left work because of the fact that I couldn’t get a test at work. The staff testing then was available through one of the drive-through centres over 20 miles from my house and quite frankly I didn’t feel well enough to drive that distance. I think staff testing would really help individuals, but it also would really help running the service, who’s got it & who hasn’t.”* (Doctor 33)  *"Generally, the response from the community has been great and we feel well supported and well looked after."* (Anaesthetist 23)  *“How stressed everyone around is… I mean I’m stressed too, I’ve never had so many nightmares in my life […]. I don’t want to talk about it, I just want to sleep more. […] You just want to sleep or have a little bit more energy for yourself, and it’s hard, and it’s going to continue being hard.”* (Anaesthetist 11) |
| **Theme 2: Concerns, unsettling experiences and difficult moments** | |
| - Anxiety and anticipation caused by: news reports on ICU services in other countries, fear of bringing the virus home, rationing of care between patients, understaffing due to sickness, uncertainty of diagnosis (lack of testing), frequent changes in PPE and clinical guidelines, moral labour of accompanying patients’ families remotely, numbers of patients requiring intensive care, numbers of patient deaths, backlog of non-COVID-19 clinical care, consequences of a second peak on patients and HCWs - Elements alleviating worries: training, experience, transparency and communication from managers | *“The patients die far too often for what you have been trained for. We are, particularly as anaesthetists working in intensive care, [...] So psychologically it’s horrible […], palliative care doctors, are used to that, decide to do that, but also they do that because they can accompany these people, they try to give them the best death possible. This is horrible, it’s away from the family, the family cannot hug them, cannot be with them.”* (Anaesthetist 11)  *“Visitors that cannot see the dying patients is the hardest part. Occasionally we have been letting family members visit but that is really hard.”* (Doctor 20)  *“Staff morale is pretty good so far, in a few weeks, it’s gonna be really demoralizing with more deaths. Normally we wouldn’t have end of life patients every day but now there could be a lot of it. It’s especially hard when visitors can’t come in and patients are dying when you’re on a really busy shift.”* (Nurse 21)  *“The other really big impact of this is on that doctor patient interaction and also that doctor relative interaction. When you've got patients on the ward and they are stuck in a room on their own and everyone in the room is dressed in PPE and they can’t have their relatives visiting them that’s actually really frightening and stressful and will create problems for people. Particularly then when they are getting critically ill and we’re communicating with the family on the phone rather than seeing the family face-to-face that’s just the other aspect of managing patients with COVID which I think all the doctors and nurses have found really difficult and really challenging.”* (Doctor 33)  *“I think people are feeling quite jarred especially when they are speaking to patients about not having treatment. I think a lot of people are finding that really hard... I am not certain what the cancer patients' outcomes will be without access to treatments, I find this heart-breaking."* (Pharmacist 18) |
| **Theme 3: Experiences around PPE** | |
| - Anxiety around: correct method of PPE donning and doffing, less availability of PPE for nurses than doctors, changing guidelines on PPE use because of lack of stock rather than safety, patients’ visitors - Discomfort of PPE: one-size PPE, working in full PPE, lack of breaks, overheating, dehydration, exhaustion, PPE as a hindrance for communication with colleagues and patients | *“The other anxiety is, I am managing patients in an intensive care environment which is not an environment that I’m used to working with, I don’t know any of the people working on ICU. I don’t know the nurses, the junior doctors, I don’t know the intensivist, and none of them can communicate with me properly because I’m wearing a mask… it’s a completely different way of working that what we normally do.”* (Anaesthetist 25)  *“It’s much more difficult to communicate with the other doctors, the nursing staff and the patients […] You have to shout loudly, which is pretty bad on the night shift because obviously you don’t want to wake up the patients that might be sleeping.”* (Anaesthetist 06)  *“One week, when I was in A&E, day by day the protocol would change, or the information would change so it was very difficult to keep up with it. […] I think, earlier on, it would have been strengthened by having a dedicated team to sort through this because […] everything was kind-of word of mouth almost […]. Considering we’re going out, seeing the suspected patients with very little protective equipment, I think […] looking back it’s a bit sort-of upsetting that […] there wasn’t strong guidelines there already.”* (Doctor 13)  *“PPE provision has been enormously stressful for lots of grades of staff. Mainly because the shortages have been quite well documented and guidance on PPE on what PPE to use has sort of changed sometimes almost daily. So, I quite often would go onto the ward to go see patients first thing in the morning and would find staff confused and not sure about what PPE they should be wearing and a lot of anxiety amongst staff, so on the staff side quite a lot of anxiety.”* (Doctor 33)  *“When wearing the PPE, I go for less water breaks than I normally would, the masks are horrendously uncomfortable, and I am getting marks on the cheeks.”* (Healthcare assistant 26) |
| **Theme 4: Morale and barriers to performing confidently** | |
| - Morale remained high – Positive feelings linked to fast and efficient pace of service changes, responsive senior management, empowerment of junior doctors and nurses - Concerns that morale will deteriorate over time because of strenuous working conditions - Barriers to performing confidently: lack of sleep (staff sickness cover and night shifts), largest work increase for ICU nurses, inability of providing highest quality of care, changes in role, working with new colleagues, PPE | *“People come across as a bit anxious and nervous but, in general, there is a good sense of camaraderie.”*  (Nurse 21)  *“I think most trainees feel that it is very easy to make suggestions on how things can be improved, and I think the more senior clinicians and management team have been quite receptive have taken many of these on board.”* (Anaesthetist 05)  *“There’s definitely anxiety or worrying about catching covid. We hear stories of healthcare workers that have died from covid. It definitely has induced anxiety. Having said that, in general, the feeling has been – I don’t want to say positive – but there’s a kind of ‘can-do’ attitude, ‘this is crisis time, this is war time, we need to get stuff done’. So, from that point of view I feel that morale has not been – even though there are these things to worry about and maybe some anxiety around PPE and Covid-19 patients – generally morale has been pretty good. And I think that people are just taking in the strain, they just wanting to do a good job and try to see this thing through. How that progresses over the weeks to months to come…”* (Anaesthetist 08)  *“Morale is a strange one. There is a lot of appreciation for the NHS (like the clapping) but also lots of people are off sick and worried. Some are putting a front on, saying ‘we’ll get it through it together’, but are still worried.”* (Nurse 22)  *“Going into the night shift with all the emergency bells ringing and not even knowing my way around ICU, that was very very challenging. I may have shed a tear on my first night shift.”* (Anaesthetist 25)  *"Lots of people are working at night, which is more difficult than in the day, and your performance is just not the same at night as it is in the daytime."* (Anaesthetist 29)  *"The intensive care nurses, they are spread very thinly, and they are very busy. So, I'd say they are probably the most stressed out people in this whole disaster."* (Anaesthetist 28) |
| **Theme 5: Life outside the clinical role** | |
| - Concerns outside clinical role: difficulties securing childcare due to fear of infection, navigating caring duties of children and elderly relatives, completing shopping and housework, travelling to work, lack of cleaning facilities at work to prevent contamination, lack of break space, keeping a life-work balance (increase workload and limited leisure options) - Relief provided by: supportive families, sustained schooling, supportive managers, free parking and accommodation - Grateful for community support - HCWs living alone found isolation difficult and some reported doing significantly more clinical work to cover for colleagues with families | *“Our childminder […] decided 2 weeks ago that because we are healthcare workers they didn’t want to look after my kids anymore. The school then shut […]. I had a text from one of the other mums at the nursery, […] she wasn’t sure she would want her child to go in view of the fact my other half and I both work at the hospital. Both of these events really affected me and my ability to focus at work.”* (Anaesthetist 06)  *"You want a shower before you go home and if you don’t have facilities to shower… you don’t want to go home to your children and give it to them."* (Anaesthetist 11)  *"It makes a massive difference being able to drive to work.”* (Anaesthetist 25)  *“I live on my own and some of the isolation policies are a bit annoying because you feel like people who are isolating because a member of their family is sick… Well you kind of, you know, feel that you’re having to cover for those people sometimes, but at the same point it isn’t the same as covering for someone who is actually sick.”* (Anaesthetist 07)  *“I think having somebody at home is very useful. I think people who live by themselves are finding it very difficult, because they are having to think ahead and make sure to prepare food on their days off so that, on their days on, they have something prepared in the fridge or the freezer.”* (Anaesthetist 14) |
